# Morton Filter-Based Security Mechanism for Healthcare System in Cloud Computing

**DOI:** 10.3390/healthcare9111551

**Published:** 2021-11-15

**Authors:** Sugandh Bhatia, Jyoteesh Malhotra

**Affiliations:** 1 Department of Computer Science, Faculty of Engineering and Technology, Guru Nanak Dev University, Amritsar 143005, India; 2 Department of Engineering and Technology, GNDU Regional Campus, Jalandhar 144007, India; jyoteesh@gmail.com

**Keywords:** COVID-19, cloud computing, digital artifacts, Morton filter, E-health records

## Abstract

Electronic health records contain the patient’s sensitive information. If these data are acquired by a malicious user, it will not only cause the pilferage of the patient’s personal data but also affect the diagnosis and treatment. One of the most challenging tasks in cloud-based healthcare systems is to provide security and privacy to electronic health records. Various probabilistic data structures and watermarking techniques were used in the cloud-based healthcare systems to secure patient’s data. Most of the existing studies focus on cuckoo and bloom filters, without considering their throughputs. In this research, a novel cloud security mechanism is introduced, which supersedes the shortcomings of existing approaches. The proposed solution enhances security with methods such as fragile watermark, least significant bit replacement watermarking, class reliability factor, and Morton filters included in the formation of the security mechanism. A Morton filter is an approximate set membership data structure (ASMDS) that proves many improvements to other data structures, such as cuckoo, bloom, semi-sorting cuckoo, and rank and select quotient filters. The Morton filter improves security; it supports insertions, deletions, and lookups operations and improves their respective throughputs by 0.9× to 15.5×, 1.3× to 1.6×, and 1.3× to 2.5×, when compared to cuckoo filters. We used Hadoop version 0.20.3, and the platform was Red Hat Enterprise Linux 6; we executed five experiments, and the average of the results has been taken. The results of the simulation work show that our proposed security mechanism provides an effective solution for secure data storage in cloud-based healthcare systems, with a load factor of 0.9. Furthermore, to aid cloud security in healthcare systems, we presented the motivation, objectives, related works, major research gaps, and materials and methods; we, thus, presented and implemented a cloud security mechanism, in the form of an algorithm and a set of results and conclusions.

## 1. Introduction

The availability of medical records, in the digital form, has played a significant role during the first wave of COVID-19. There was no medicine and vaccine during the first wave of this deadly virus in the year 2020. The only way to combat this zoonotic virus was to maintain social distance. During the second spike of COVID-19, in the month of June 2021, when millions of people had been administering jabs of vaccine every day worldwide, the most prominent method to control this virus was social distancing. Many experts have designed machine learning algorithms to detect the coronavirus in a suspect. After the confirmation that the person is suffering from coronavirus, consultation with the qualified medical practitioner is required. Cloud computing has performed and is still performing a prominent role in the area of E-health services during this pandemic. The area of applications of cloud computing has been widening day by day. It is considered one of the most cost-efficient computing paradigms. At the same time, researchers and scientists criticized the cloud ecosystem [1] and services, due to the lack of digital forensic tools and technologies applied in the cloud computing environment. No technology or paradigm of computing can be declared as a 100% fool-proof secure system. This poses that the privacy and security of patient’s information and records must be ensured. Digital medical transcripts are of great importance, as the overall diagnosis is dependent on these reports, and any tampering with the transcripts might be life threatening. To investigate any tampering or modification in the patient’s data, digital forensic techniques must be used. Cloud forensic investigation [2] is of the prime concern today, due to the availability of sensitive data, such as medical and financial on the cloud. There is extreme necessity to take immediate action, such as seizing artifacts quickly, acquiring the evidence, and analyzing the dossier in a timely manner. There is a possibility that digital artifacts may be tampered or modified while transferring from cloud infrastructure to the cloud forensic investigation team. In addition, the seized digital artifacts must be reliable and impeccable. It is the need of the hour to design a framework [3] to ensure the reliability of these seized digital artifacts. The present article focuses on the development of a mechanism, which is specifically designed to point out the location of modified or altered artifacts, while moving from the cloud infrastructure to the forensic investigation team. In this article, we represent a space-efficient scheme, which is specifically designed to find out the position of modified digital artifacts. The principal advantage of this mechanism is that it can be comfortably integrated into various available forensic transmission schemes. Furthermore, this designed mechanism is used to locate the position of tampered data in a hierarchical block. To achieve this, a novel algorithm is designed, with the help of the Morton filter [4]. The major property of this system is that it is based upon space-efficient probabilistic data structure, which can be applied to verify whether an element belongs to a specific set or not. The Morton filter is selected, due to its high throughput, when compared with other probabilistic data structures, such as cuckoo, semi-sorting cuckoo, and rank and select quotient filters. Another data structure that is used to store the metadata at the application level is stack. When the modified digital artifact is detected, the proposed algorithm can promptly locate it and reveal its position in the hierarchical block.

The rest of this paper is organized as follows. Section 2 describes the motivation, main objectives of the proposed mechanism, related works, and major research gaps. Section 3 explains the mechanism design, along with explanation of symbols used in the mechanism. Section 4 introduces the working of the system and introduction of designed algorithm. Section 5 explains the functionality of the algorithm. Section 6 highlights the benefits of using the Morton filter. Section 7 presents the performance evaluation and results. Finally, we give concluding remarks in Section 8.

## 2. Motivation

In cloud-based healthcare systems, the treatment of the patient is dependent upon digital medical transcripts. The reliability and accuracy of these medical transcripts must be ensured during the transfer of E-health records from patient to doctors. Cloud forensic is a prominent digital forensic technique that can be applied to perform examinations in cloud-based systems. The result of forensic analysis is dependent upon the digital artifacts. The reliability of digital artifacts is one of the most important objectives to achieve in the entire forensic process. When the job of collection of artifacts is over, then the phases of identification, collection, preservation, analysis, examination, and presentation start. Even after the completion of analysis, all the artifacts must be preserved [5]. Any alteration or tampering of digital artifacts will break the chain of custody. In each service model of cloud computing, forensic investigators have demonstrated the procedure to perform live forensics. There are various challenges that revoke the live cloud forensics in real applications, and we are discussing some of them. When digital artifacts are seized from the channel, it must be promptly transferred to the forensic analysts. If it is required to retransfer the altered or tampered data [6], then it must be ensured that only the part of altered data or artifact is transferred and not all of the data. In such a way, it is economically feasible for forensic analysts to find out the position of tampered digital artifacts. Another major problem in sending the digital artifacts as streaming data packets is the absence of metadata and other semantic values. It is a tedious and complex task to relocate the corrupted or altered data within a hierarchical block of artifacts during real-time analysis. It is possible to transfer raw data and metadata simultaneously to handle the problem, but it will not be economically feasible for the organization. Moreover, it will slow-down the entire process, and redundancy in the metadata can occur at some level. Therefore, an effort has been made to develop a mechanism to find the location or position of altered, modified, and tampered data in a block of data hierarchy. The fundamental motivation of this mechanism is that it specifies the position of tampered data within a block of data stream [7]. This mechanism takes the benefits of the Morton filter [8] and a stack [9] to place and fetch the metadata on the application level. When the altered data is identified, the mechanism can restrain it immediately and conveys its position in the hierarchical block. Hence, this space-efficient mechanism can be applied to ensure the reliability of digital artifacts in cloud forensics [10] for cloud security [11].

### 2.1. Objectives of the Mechanism

The principal objectives of the proposed security mechanism are given below.

- Various secure, cloud-based healthcare systems were designed with probabilistic data structures, such as cuckoo [12], bloom, and attribute bloom filters. The most advanced and efficient filter is the Morton filter, and it is the best approach to design a cloud security mechanism with Morton filter as throughput, when compared with other probabilistic data structures.
- Designing a security mechanism that provides secure data storage in cloud-based healthcare systems.
- Employing fragile watermark that has the capability to find any tampering of data and digital artifacts.
- Designing a generic security mechanism that can be applied to various cloud-based healthcare systems.
- Realizing a performance evaluation of the proposed security mechanism, based on metrics such as throughput for lookups, insertions, and deletions load factor, but also by making a security and privacy analysis and assessment of the designed security mechanism, by comparison with other similar approaches.

### 2.2. Related Works

Various data structures have been used over the years by researchers to accomplish the task of tamper detection. A non-binary Merkle tree can be used in case of a hierarchical dataset whenever there is a limited number of operations. This method has been applied only for the case of hierarchical data and is not applicable for streaming data. Code self-checksumming has been implemented to detect static and dynamic patches, delivered by potentially unwanted programs (PUP) [13]. This tamper detection mechanism was able to checksum instruction with the combination of absolute address and relocation. Proof of past data possession (PDDP) [14] contains the data possession proof to check whether the user originality possessed the evidence or not. As per this mechanism, it is possible for the user to delete the records from the storage, but PDDP modification is not possible. Bloom filter storage was used in this mechanism. Tamper evident logs were used for forensic purposes. This method was based upon regular and continuous auditing. Tamper evident log design was completely dependent on the Merkle tree, and it allows a logger to furnish the proof of its functioning. Cryptographic, one-way hash functions were used to develop the mechanism. Database management system stores the audit logs. Authors explored a novel idea to provide audit logs to detect tampering effectively in transaction processing systems. The cuckoo filter has been used to develop a fast tamper detection mechanism for the hierarchical data in cloud forensic. The authors claimed that the designed mechanism has full compatibility with existing forensic data transmission schemes. The bloom, cuckoo, and counting bloom filters have been implemented to verify the reliability of digital artifacts. In barcode recognition and processing [15], the bloom filter performed a significant role. The single-hash lookup cuckoo filter [16] was used, with one hash function, to find out any specific item. Redundant records can be identified by implementing the accurate counting bloom filter [17]. Remya et al. [18] presented a mechanism for the security of critical electronic health records (EHRs). In this mechanism, security can be achieved with the support of identity-based secure and encrypted data-sharing techniques. Multiple solutions were suggested to secure cloud-based data and records in e-health systems. Security threats and attacks [19] in cloud computing were discussed in detail. Various types of machine learning algorithms were investigated and implemented as a tool to provide security in cloud computing applications. Secure and efficient digital data sharing system for cloud environments [20] have been extensively developed and scholars have presented a cloud-based, student-centered mechanism to solve the problem of user management. The system was based on Lagrange interpolating polynomials. To secure software-defined networks (SDNs) [21] from threats and attacks, a system was proposed that utilizes the benefits of software-defined networks characteristics, along with data mining, to find any malicious activity in the data plane of SDN. Qiang et al. [22] explained the role of digital technologies to control the coronavirus spread. Digital technologies, such as cloud computing, artificial intelligence, internet of things, wearable devices, blockchain technology, and 5G communication networks, have played an irreplaceable role with public health systems in controlling the second wave of COVID-19 in China. Cloud and network security was achieved by applying network and cloud forensics, in association with software-defined networking. Recommendations were given by the authors to apply security and privacy in cloud forensic by using static, dynamic, and remote cloud forensics by Quadri et al. [23]. Shangbin et al. [24] demonstrated a framework that can be used to eliminate the effects of noise and anomalies by implementing robust online evolving anomaly detection (ROEAD) and a robust feature extractor (RFE). A comprehensive analysis, in the area of cloud computing, enabling the internet of things was performed. Privacy protection models, frameworks, and methods were explored. Key challenges, threats, and attacks in cloud computing security were discussed by Tahirkheli et al. [25]. A security and privacy-preserving mechanism was developed to maintain the integrity and confidentiality in electronic health records of patients. It is economically feasible to store, process, and update the electronic data of patients in cloud computing [26]. Graph models [27] were developed and tested to provide security in cloud computing. Cryptographic threshold techniques were applied, along with intelligent secret management and data sharing techniques. Seh et al. [28] discussed the simple moving average and simple exponential smoothing methods, in order to investigate data breaches in healthcare systems. Results of the time series analysis revealed that the cost of data breaches and number of data breaches will increase in near future. Liu et al. [29] proposed a system based on blockchain and distributed ledger-based improved biomedical security system (BDL-IBS), in order to strengthen privacy and security in healthcare applications. Chadwick et al. [30] designed an architecture for sharing the cyber threat information. It is a five trust model, which is based upon five levels. Data sharing agreements (DSA) and data protected objects (DPOs) are important constituents of the architecture, and these constituents perform a significant role in the successful implementation of cloud-edge based data security architecture.

### 2.3. Major Research Gaps

The related work and research studies draw an intense observation that security and privacy for healthcare systems in cloud computing is enormously challenging. Thus, we are able to find significant gaps and shortcomings of previous studies. A preliminary review of the research literature reveals that, despite the abundance of work in the area of cloud-based healthcare system security and privacy in electronic health records, there is a need for studies that can present the findings and analyze the limitations of previous studies, in terms of probabilistic data structures, such as the cuckoo, bloom, semi-sorting cuckoo, and rank and select quotient filters. To identify the research gaps, articles between 2016 and 2021 were analyzed. Table 1 summarizes work on the cloud-based healthcare system security and privacy of electronic health records, through various probabilistic data structures. As shown in Table 1, the majority of the security mechanisms are designed on bloom and cuckoo filters. None of the mechanisms were designed on the basis of Morton filter, which is considered one of the most efficient probabilistic data structures [8]. Filling this gap, we have designed a Morton filter-based security mechanism for healthcare systems in cloud computing. Another major gap is that no cloud security mechanism has been developed with the integration of least significant bit replacement watermarking and class reliability factors. Our proposed security mechanism, for the healthcare system, is developed with least significant bit replacement watermarking. No cloud security system is capable of performing the reliability test on digital artifacts in cloud forensics. Our proposed mechanism is capable of performing reliability tests on the acquired digital artifacts.

In this research work, the Morton filter has been used, due to better insert, delete, and lookup throughput. The Morton filter [8] is compared with the cuckoo, semi-sorting cuckoo, and rank and select quotient filters, as all these are space-efficient probabilistic data structures. These data structures can be implemented to check that a particular element is a member of a set or not.

## 3. Discussion

The study proposes a new Morton filter-based security mechanism for healthcare systems in cloud computing to provide the security and to maintain the integrity of electronic health records. The most important issue, which is resolved in this research article, is how to find out the position of altered data or artifacts from the hierarchical block. A block can be defined as B = {b_1_, b_2_… b_n_}, where b_i_ (1 ≤ i ≤ n) is an indivisible data constituent available in the block. The data constituent can be further divided into the p data class, such as B = {C_1_, C_2_… C_p_} (1 ≤ r ≤ p). A data class C_r_ can be demarcated if, and only if, its last constituent $b_{0}^{r}$ of class C_r_ can be considered as the boundary integral element of C_r_. While acquiring the digital artifacts, a hierarchical block structure can be generated as a tree. A class of data entities, such as a records, can be formed as an internal node of a tree, whereas a slice of data element can be taken as a terminal node of the tree. Moreover, along with data class C_r_, another significant class is described as the demarcated class C_d_, in this C_d_ (value = Hash (fdr_i_) (1 ≤ i ≤ 0) C_d_.group ∈ { “€”, “£” }). Presented as a block of data in the form of a tree that is traversed with iterative deepening bidirectional search [45]. Two inputs are given in C_d_ and in a hierarchical block, one is “€”, which is inserted before an internal node is traversed; the second is “£”, and it is inserted when a terminal node has been traversed.

**Figure 1:**
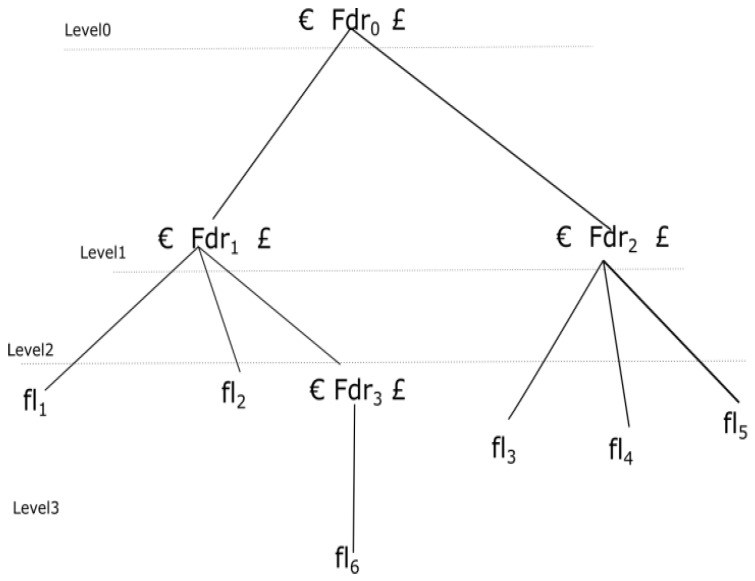
Traversal operations on hierarchical block. Fdr: Directory; fl: File; €: inserted into the data stream before a non-leaf node; £: inserted into the data stream after a non-leaf node has been traversed.

Figure 1 illustrates the demarcated class and the moment of inputting into the tree. Nodes will be traversed in the following order: Fdr_0_, Fdr_1_, fl_1_, fl_2_, Fdr_3_, fl_6_, Fdr_2_, fl_3_, fl_4_, fl_5_. The designed tree depicts that the internal nodes are Fdr_0_, Fdr_1_, Fdr_2_, Fdr_3_. Hence, a demarcated class of “€” is inserted. Similarly, a demarcated class of “£” is entered after the traversing of their sub-trees.

**Figure 2:**
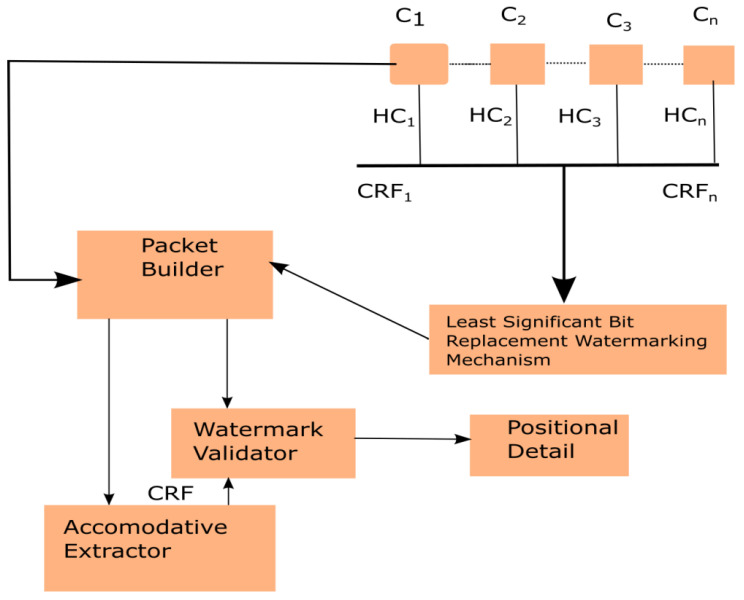
Working of digital artifacts reliability system. HC: Hash value of class; C: Class; CRF: Class reliability factor.

Figure 2 shows the flow of operations in the designed mechanism, which executes the system and consists of five major constituents: LSB replacement watermarking mechanism [46], accommodative extractor [47], watermark validator [48], packet builder, and positional details. The transmitting data is classified into data classes C_i_ (i = 1, 2 … n). The hash values of class C_i_ are calculated as HC_i =_ H(C_i_) (i = 1, 2 … n), where H is taken as a hash function [49]. Two contiguous class hash values are grouped and computed simultaneously as H (H(C_i_) || H (C_i+1_), it is denoted as the class reliability factor (CRF). The dependability in CRF can be achieved with the private key [50] is given by the sender. The CRF will be implanted, such as fragile watermarks [51], within the packets. Fragile watermarks have the capability to detect any modification or tampering of data. After the delivery of the data packet, the accommodative extractor initially extracts the watermark associated with that packet.

At the same time, raw data is required to calculate CRF, and if the value of calculated CRF matches with the extracted watermarks, then it will be considered that the data class is without any alteration or tampered data; else, the class will be treated as an altered class. For more details, regarding the algorithm for the detection of malicious activity, watermarking as a service (WaaS) [52] can be used.

## 4. Materials and Methods

After inputting the demarcating class in the data stream, the location of altered or tampered data can be found from the hierarchical block (tree). Various well-discussed data structures are available that can be used for the purpose of designing the data block, such as tree, hash map [53], block chain, and link list. The primary glitch in all these data structures is the requirement of more and more space when private keys are attached with the data structure. Therefore, it is significant to use a space-efficient probabilistic data structure [54] for the storage of internal nodes. The Morton filter, an approximate set membership data structure, is used to overcome the problem. It is an improved data structure, when compared with bloom [55] and cuckoo filters [56], in terms of space-efficient insertion, deletion, and lookup. An algorithm named as identify_positions (Algorithm 1) has been designed, and it explains the algorithm that can detect the position or location of modified or tampered data in the hierarchical block. Four inputs are given: an uninitialized Morton filter (MF), a data stream (DS), an empty stack (TK), and a counter initialized as 0. It provides the result as a set of modified positions (MPS). Once the modified positions are detected and available, it is possible to fetch modified or altered data from MPS. Different subroutines are incorporated in the algorithm discussed below. The subroutine initial is used to initialize the data structure of MF and TK. Another subroutine, chkcls, verifies the number of classes available in streaming data. The subroutine ensures whether MF has a private key. The subroutines inst and del perform operations to insert and delete the key from the MF. Other routines, such as pop, push, peek, and stacktraceback, represent the basic operations of stack data structure.
**Algorithm 1**. Identify positions.1. Procedure: identify_positions of the modified data2. **Start**3. **Input:**
*The data class C_i_ in the data stream DS, the Morton Filter MF, Stack TK, counter i.*4. *DS*
*← ø*
*; initial (MF), initial (TK);*5. *i ← 0*6. **while**
*C_i_ ← DS.chkcls ( )*
**do**7. *i++;*8. *switch C_i_.type*
**do**9. *Case “ €”*10. *h ← DS (C_i_)*11. **if**
*MF.involve (DS_i_) = = false*12. **then**
*DS.push (h);*13. *MF.inst (h);*14. *Case “£”*15. **If**
*MF.lookup (DS_i_) = = true*16. **then**
*h ← DS.pop ( );*17. *MF.del (h);*18. *Case “Data”*19. **If**
*detect (C_i_) = = true*20. **then**
*Temp_DS = peek (TK)*21. **if**
*MF.involve (Temp_D) = = true*22. **then**23. *D_j_ = stacktraceback ( );*24. *MPS = MPS*
*∪*
*{D_j_}*25. **End**Output: The set of modified positions MPS and modified data is detected.

## 5. Functionality of Algorithm

**Figure 3:**
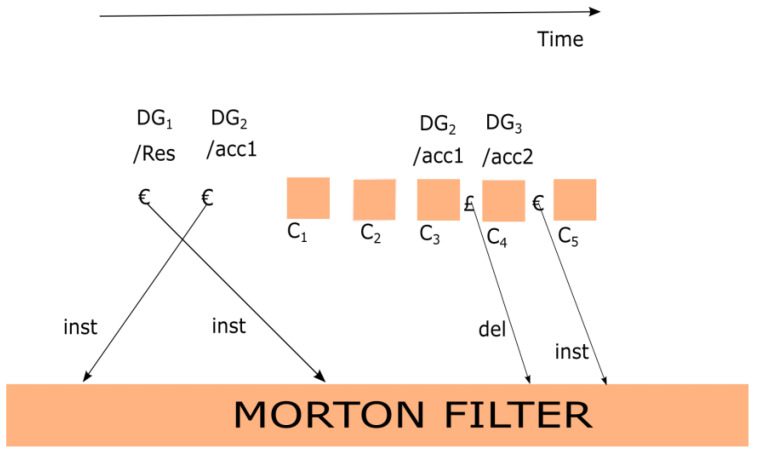
Morton filter and operations. DG: Demarcating group; inst: insert; del: delete; C: Class; DG: Demarcating groups; Res: Folder; acc1: folder; acc2: folder; €: inserted into the data stream before a non-leaf node; £: inserted into the data stream after a non-leaf node has been traversed.

The functionality of the algorithm is discussed here, with the help of an illustration drawn in Figure 3. The streaming data has been distributed into the classes C_1_, C_2_, … C_n_. The demarcating groups are DG_1_, DG_2_, and DG_3_, which are associated with the folders “/Res”, “/Res/acc1”, and “/Res/acc2”. In case of demarcating the group “€”, it will be given to the MF. Subsequently, the hash of a similar class will be pushed into the TK. On the other hand, if the demarcating group is “£”, it will be wiped out from the MF and from TK, and the hashed will be popped out. The detection algorithm [57] is capable of finding any alteration or tampering within the classes C_1_, C_2_, … C_n_.

The value at the top of the stack will be fetched and then checked against MF. If MF holds the value, the stack trace will be included into the set of positions (PS) from where the modified data was detected. If class C_2_ is detected as a modified class, then DG_2_ will be recovered from the top of stack. As it has been injected into MF, the subroutine stacktraceback will be executed and placed at the location “/Res/acc1” into MPS. On the same note, the modified class C_3_ will perform the same operations on MPS, and MPS will be constant. When modified class C_4_ will be detected, the stack trace will hold the value of DG_1_; hence, DG_1_ is the only value in the stack, and the location “/Res” is included into the MPS. Finally, the modified class, C_4_, will identify that DG_1_ and DG_3_ are in TK. Therefore, “/Res/acc2” will be inserted into the MPS. As the outcome, the position of the modified class in the hierarchical block and data class will be provided by the designed mechanism.

## 6. Benefits of Implementing the Morton Filter

**Figure 4:**
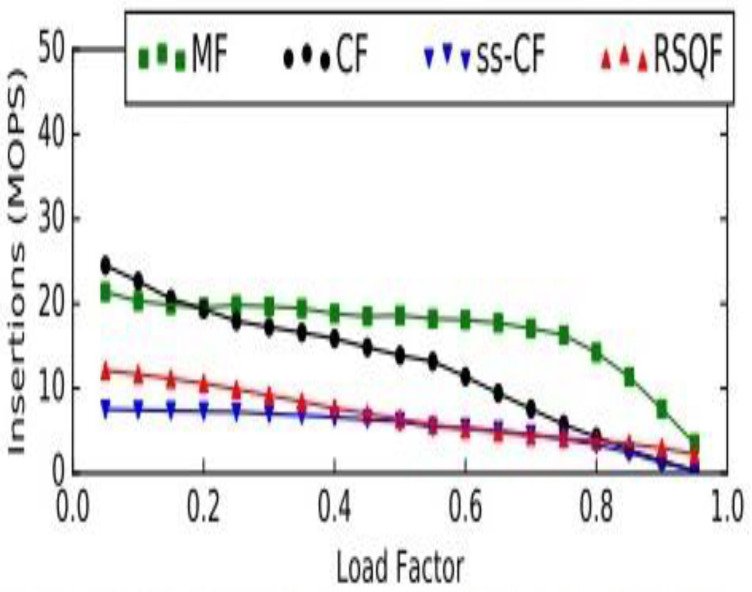
Comparison between Morton and cuckoo filters for insert operation. MOPS: millions of operations per second; MF: Morton filter; CF: Cuckoo filter; ss-CF: semi-sorting Cuckoo filter; RSQF: rank and select Quotient filter.

The complexity of the procedure identify_positions is linear to the number of classes. For each class, the time complexities of various operations, such as insert, delete, and lookup, are *O* (1). Moreover, time complexity for basic stack operations, such as push and pop is *O* (1), as it is possible to work at only one end of this linear data structure. The cuckoo filter [58] is considered a better option than the bloom filter in three ways. It supports the dynamic deletion of items. It has better lookup performance, and, most importantly, it has better space efficiency in applications that require low false positive rates (є < 3%). However, a Morton filter is an improvement to cuckoo filters. It is called a modified cuckoo filter. It supports various operations, such as insertion, deletion, and lookup, as cuckoo filters. Additionally, it achieves a high throughput and quotient filter, which enhances the capability of the Morton filter, by leveraging its internal representation. It also requires less memory than the cuckoo filter for a similar error rate. Its insert throughput is 15.5×, delete throughput is 1.3×, and lookup throughput is 2.5× higher, when compared to cuckoo filter. A comparison is made in the Figure 4 between the Morton, cuckoo, semi-sorting cuckoo, and rank and select quotient filters. The comparison reveals that an MF is approximately 0.9× to 15.5× faster than a CF for insertions.

**Figure 5:**
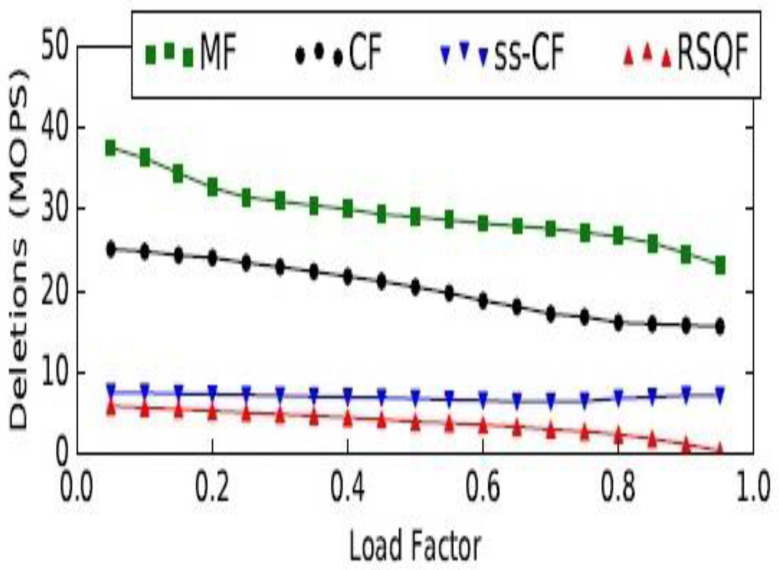
Comparison between Morton and cuckoo filters for delete operation. MOPS: millions of operations per second; MF: Morton filter; CF: Cuckoo filter; ss-CF: semi-sorting Cuckoo filter; RSQF: rank and select Quotient filter.

**Figure 6:**
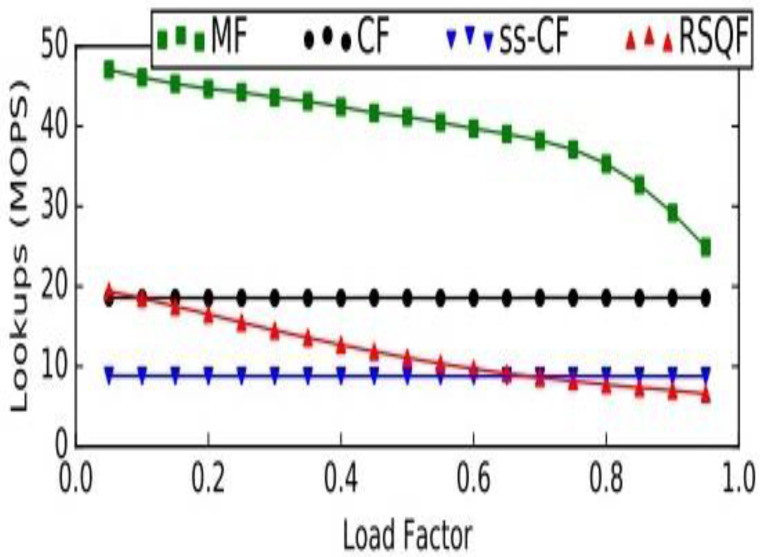
Comparison between Morton and cuckoo filters for lookup operation. MOPS: millions of operations per second; MF: Morton filter; CF: Cuckoo filter; ss-CF: semi-sorting Cuckoo filter; RSQF: rank and select Quotient filter.

Similarly, other operations are depicted in the Figure 5 and Figure 6, respectively, regarding the delete and lookup operations in MF, CF, ss-CF, and RSQF. In delete operation, also, the MF is faster, by 1.3× to 1.6×, than the CF.

## 7. Results

Experiments were conducted on three servers with a 2.5 GHz Intel i5 10th generation CPU and 8 GB RAM (Lenovo, Beijing, China). We used Hadoop version 0.20.3 (Apache Software Foundation, Forest Hill, MD, US), and the platform was Red Hat Enterprise Linux 6 (Red Hat, Inc., Raleigh, NC, US). We executed five experiments, and the average of the results was taken. The proposed mechanism exhibits space efficiency. Total numbers of 100,000 bits were taken and accommodated 10,000 different constituents of metadata with a false positive rate of 7.496 × 10^−12^. However, the mechanism can organize 805 different metadata pieces, in the case of the implementation of the SHA-256 hash function. In other situations, it is possible to organize 1610 metadata constituents, if the MD5 hash function is implemented. The experiments initially analyzed a false positive rate [59] of the Morton filter, with the increasing quantity of metadata. A comparison is performed between the false positive rate of the Morton and cuckoo filters. The details of both the Morton and cuckoo filters are given below in Table 2 and Table 3.

The Morton filter is an improvement over the cuckoo filter in three ways.

1. Memory utilization is one of the important advantages of using the Morton filter.
2. It is placed in a better position, when compared to the cuckoo filter, in the context of insert, delete, and lookup [60] throughput.
3. Its implementation is not a complex and tedious task, as in the case of earlier filters, such as the bloom, quotient, and cuckoo filters.

Therefore, the proposed that a digital artifacts reliability system can be implemented as a security mechanism, in order to ensure the reliability of electronic health records and medical transcripts in cloud-based healthcare systems, and it can be used to strengthen the cloud forensics [61] process. An effort has been made by the authors to overcome all the limitations and drawbacks of bloom, quotient, and cuckoo filters, by using the Morton filter.

## 8. Conclusions

In this research paper, we discuss the design and implementation of a Morton filter-based security mechanism for cloud-based healthcare systems, which can provide security and privacy to electronic health records or digital medical transcripts. In contrast to the existing schemes, the developed mechanism uses the Morton filter for storage, and it can be applied in the area of cloud forensics. For the successful implementation and execution of the discussed mechanism, a couple of algorithms and forensic data transmission schemes are integrated with this system. Tampered digital artifacts and their location can be pinpointed in real-time. In addition, there are many options for probabilistic data structures, such as cuckoo, bloom, semi-sorting cuckoo, and rank and select quotient filters. Compared to the cuckoo filter, this Morton filter-based security mechanism is lighter and has less computational overhead, with time complexity *O (1)* and load factor 0.9, in finding the location of altered or tampered data in hierarchical data block in cloud-based healthcare systems. Hence, it permits the digital forensic team to localize the tampered or altered data and its location promptly. For further studies, we intend to apply advanced hashing techniques, along with other probabilistic data structures.

## Figures and Tables

**Table 1:** Security and privacy mechanisms, designed with probabilistic data structures.

Reference	DataStructure/ Technology Used	Cloud-Based Healthcare System	Contribution
Ying et al. (2021) [31]	Cuckoo Filter	No	Suggested a security-enhanced attribute cuckoo filter to hide the access policy and designed ciphertext-policy attribute-based encryption
Xie et al. (2021) [32]	Cuckoo Filter	No	Proposed a lattice signature method, with Cuckoo filter, that can simplify the computational overhead
Kumar et al. (2021) [33]	Bloom Filter	Yes	Explained technique to protect cloud datasets with bloom filter, based ciphertext-policy attribute-based encryption
Cano et al. (2020) [34]	Elliptic Curve Cryptography	Yes	Presented a solution to achieve security and the preservation of data privacy in internet of medical things and the cloud
Breidenbach et al. (2020) [35]	Bloom Filter	No	Discussed privacy-preserving concept, by using bloom filter and cryptographic functions
Shi et al. (2020) [36]	Block Chain	Yes	Investigated various approaches of E-health records in blockchain technology and proposed different applications of healthcare in blockchain
Adamu et al. (2020) [37]	Laravel Security Features	Yes	Proposed a framework that can be used to apply security and privacy to electronic medical record
Breslow et al. (2019) [8]	Morton Filter	No	Designed a mechanism to prove that the Morton filter is an improvement over the cuckoo filter
Jeong et al. (2019) [38]	Bloom Filter	No	Proposed a secure cloud storage service, on the basis of bloom filter and provable data possession model
Patgiri et al. (2019) [39]	Bloom Filter	No	Explored the adaption of bloom filter in network security, packet filtering, and IP address lookup.
Ming et al. (2018) [40]	Cuckoo Filter	Yes	Designed an attribute-based signcryption scheme (ABSC) for privacy-preserving in electronic health record
Decouchant et al. (2018) [41]	Bloom Filter	No	Presented a bloom filter-based novel filtering method that can be applied to reads of any length
Ramu (2018) [42]	Attribute Bloom Filter	Yes	Proposed a secure cloud mechanism to share health records among various users, using ciphertext-policy attribute-based encryption and attribute bloom filter
Brown et al. (2017) [43]	Bloom Filter	Yes	Discussed privacy-preserving record linkage (PPRL) model, along with bloom filter, to overcome the problems of data integration and privacy
Vatsalan et al. (2016) [44]	Counting Bloom Filter	Yes	Proposed a novel method to provide privacy for multi-party privacy-preserving record linkage with counting bloom filter

ABSC: attribute-based signcryption scheme; PPRL: privacy-preserving record linkage.

**Table 2:** Parameters used by cuckoo filter.

Token and Its Explanation	α (Load Factor)	m (No. of Buckets)	b (No. of Entries per Bucket)	f (Length of Fingerprints in Bits)
Value	0.8	8	8	36

**Table 3:** Parameters used by Morton filter.

Token and Its Explanation	α (Load Factor)	m (No. of Buckets)	b (No. of Entries per Bucket)	f (Length of Fingerprints in Bits)
Value	0.9	8	8	36

## Data Availability

Not applicable.
